# Hazardous Continuation Backward in Time in Nonlinear Parabolic Equations, and an Experiment in Deblurring Nonlinearly Blurred Imagery

**DOI:** 10.6028/jres.118.010

**Published:** 2013-04-24

**Authors:** Alfred S Carasso

**Affiliations:** National Institute of Standards and Technology, Gaithersburg, MD 20899

**Keywords:** advection dispersion equation, backward parabolic equations, hydrologic inversion, image deblurring, ill-posed continuation, non-uniqueness, Van Cittert iteration

## Abstract

Identifying sources of ground water pollution, and deblurring nanoscale imagery as well as astronomical galaxy images, are two important applications involving numerical computation of parabolic equations backward in time. Surprisingly, very little is known about backward continuation in *nonlinear* parabolic equations. In this paper, an iterative procedure originating in spectroscopy in the 1930’s, is adapted into a useful tool for solving a wide class of 2D nonlinear backward parabolic equations. In addition, previously unsuspected difficulties are uncovered that may preclude useful backward continuation in parabolic equations deviating too strongly from the linear, autonomous, self adjoint, canonical model.

This paper explores backward continuation in selected 2D nonlinear equations, by creating fictitious blurred images obtained by using several sharp images as initial data in these equations, and capturing the corresponding solutions at some positive time *T*. Successful backward continuation from *t=T* to *t* = 0, would recover the original sharp image. Visual recognition provides meaningful evaluation of the degree of success or failure in the reconstructed solutions.

Instructive examples are developed, illustrating the unexpected influence of certain types of nonlinearities. Visually and statistically indistinguishable blurred images are presented, with vastly different deblurring results. These examples indicate that *how* an image is nonlinearly blurred is critical, in addition to the amount of blur. The equations studied represent nonlinear generalizations of Brownian motion, and the blurred images may be interpreted as visually expressing the results of novel stochastic processes.

## 1. Introduction

This paper presents an effective iterative procedure that can be used to solve a wide class of 2D nonlinear parabolic equations backward in time. However, previously unsuspected difficulties are also uncovered that may preclude useful backward continuation in parabolic equations deviating too strongly from the linear, autonomous, self adjoint, canonical model. Instructive 1D examples of ill-behaved continuations were previously reported in [6].

Continuation backward in time in parabolic equations is a notoriously ill-posed problem with some intriguing applications. Of major current interest are *hydrologic inversion* and *image deblurring*. Hydrologic inversion seeks to identify sources of groundwater pollution by backtracking contaminant plumes, [2–5,15,18]. This involves solving the advection dispersion equation (ADE) backward in time, given the contaminant spatial distribution *g*(*x*, *y*) at the current time *T*. In image science, images blurred by Gaussian point spread functions are a common occurrence. Deblurring Gaussian blur is mathematically equivalent to solving the heat conduction equation backward in time, [8,9,16]. More recently, in [7] and references therein, striking enhancements were obtained when time-reversed fractional and/or logarithmic diffusion equations were applied in blind deconvolution of Hubble space telescope galaxy images, as well as scanning electron microscope imagery of interest in nanotechnology.

With backward uniqueness assumed to hold, prescribed *L*^2^ bounds on the solution are often used, along with smoothness and non-negativity constraints when applicable, to stabilize backward reconstruction against amplification of input data noise. These and other regularization methods have been extensively studied in recent years. However, only limited computational experience has generally been accumulated on backward problems. This is especially true for nonlinear problems in more than one space dimension. As will be seen below, difficulties may remain, even if regularization has successfully prevented noise amplification, and produced a solution satisfying prescribed bounds and other constraints.

The Van Cittert method is an iterative procedure for solving linear integral equations of convolution type, where the kernel is known explicitly and has a positive Fourier transform. The method originated in spectroscopy in the 1930’s [19], and has been used in image restoration, [11,14]. In this paper, the Van Cittert iteration is adapted into a useful tool for exploring a large class of nonlinear backward parabolic equations, for which the solution operator is neither linear nor known explicitly.

A productive setting for studying 2D backward parabolic continuation lies in the field of image restoration. One can create fictitious blurred image data, by using a given sharp image as the initial value in the nonlinear parabolic equation to be studied, and selecting the corresponding solution at some positive time *T*. Successful backward continuation from *t=T* to *t* = 0, would recover the original sharp image. An important advantage is that visual recognition can provide useful evaluation of the degree of success or failure in the reconstructed solution. This can then be translated into the original engineering context, unrelated to imaging, where backward continuation in that particular equation is of interest.

There may be deeper analytical reasons for pursuing such a program of study. Brownian motion is pervasive in many branches of science, including image science, and Gaussian blurs and the heat equation appear quite naturally in image analysis. In [7], more sophisticated blurs were contemplated, associated with Brownian motion taking place in specific randomized time, and expressed in terms of parabolic pseudodifferential equations, [10,20]. Such *subordinated* stochastic processes are of great current interest. The successful application of these notions in blind deblurring of the valuable scientific imagery discussed in [7], was unanticipated and noteworthy.

In the present paper, the nonlinear partial differential equations used to form the blurred images in Sec. 6, were chosen primarily for mathematical reasons, and may not simulate any currently known physical blur. Surprisingly, these images appear realistic, albeit with subtle differences from familiar blurred imagery. Such images may be viewed as expressing visually, the results of novel stochastic processes that are nonlinear generalizations of Brownian motion. A wide variety of nonlinearities may be explored. Sophisticated computational simulations, using high precision numerics on high resolution imagery, may yield fruitful insights into the behavior of this class of random processes. Finally, future imaging applications could well involve similar nonlinear parabolic blurs, and such exploration can help identify potential image processing roadblocks that would need to be circumvented.

## 2. Stability and Backward Uniqueness

Backward parabolic equations and other ill-posed problems are discussed in [1,12,13,17], and the references therein. In general, a backward solution may exist only for highly restricted data satisfying certain smoothness and other requirements that are not easily characterized. Typically, when a solution exists, it is unique. However, backward solutions depend discontinuously on the data for which they exist, and slight changes in these data can result in very large, if not explosive, changes in the corresponding solutions. In practice, at a given positive time, the precise data needed for the existence of a particular backward solution are seldom available, and one must use approximate values. Hence, backward stability estimates are of vital interest.

Let Ω be a bounded domain in *Rn* with smooth boundary. Let *L* be a linear or nonlinear elliptic ∂Ω operator in Ω, acting on smoothly differentiable functions satisfying homogeneous Dirichlet or Neumannn conditions on ∂Ω. Let *L* be such that the forward initial value problem *w_t_=Lw*, *t* > 0, *w*(0) = *f*, is well-posed in *L*^2^(Ω). Let *w*^1^(*x*, *t*) and *w*^2^(*x*, *t*) be any two solutions, and let $F(t)={\left\|w^{1}(.,t)-w^{2}(.,t)\right\|}_{2}^{2}$, 0 ≤ *t* ≤ *T*.

Using *logarithmic convexity* techniques, [1,12,17], the folowing inequality can be established for a wide class of parabolic equations *w_t_=Lw*,

(1) $$F(t)\leq {\left\{F(0)\right\}}^{1-\mu (t)}{\left\{F(T)\right\}}^{\mu (t)},\;0\leq t\leq T.$$

Here, the Hölder exponent *µ*(*t*) satisfies 0 ≤ *µ*(*t*) ≤ 1, with *µ*(*t*) > 0, *t* > 0, *µ*(*T*) = 1, *µ*(0) = 0, and *µ*(*t*) ↓ 0 monotonically as *t* ↓ 0. If we restrict consideration to solutions *w*(*x*, *t*) satisfying a *prescribed bound at t* = 0, i.e., ‖*w*(.,0)‖_2_ ≤ *M*, then *F*(*t*) in Eq. (1) can be made small at a given *t* > 0, by making *F*(*T*) sufficiently small.

This stabilized backward parabolic problem for *L* may be stated as follows. Given *f* (*x*) ∈ *L*^2^(Ω) and *M*, *δ* > 0, with *δ* ≪ *M*, find all solutions of

(2) $$w_{t}=Lw,\;0<t\leq T,$$

such that

(3) $${\left\|w(.,T)-f\right\|}_{2}\leq \delta ,\;{\left\|w(.,0)\right\|}_{2}\leq M.$$

It is assumed that *f* (*x*), *δ* and *M* are compatible with the existence of solutions. Here, *f* (*x*) is presumed to be a sufficiently close *L*^2^ approximation to the exact values *w*(*x*, *T*), at *t=T*, of a solution *w*(*x*, *t*) of Eq. (2), believed to satisfy ‖*w*(.,0)‖_2_ ≤ *M*. If *w*^1^(*x*, *t*) and *w*^2^(*x*, *t*). are any two solutions of Eqs. (2) and (3), the following stability inequality follows from Eq. (1)

(4) $${\left\|w^{1}(.,t)-w^{2}(.,t)\right\|}_{2}\leq 2M^{1-\mu (t)}\delta^{\mu (t)},\;0\leq t\leq T.$$

### 2.1 Backward Uniqueness

The inequality Eq. (4) implies backward uniqueness. If *δ* = 0, then ‖*w*^1^(.,*t*) − *w*^2^(.,*t*)‖_2_ = 0 for every 0 < *t* ≤ *T*, since *µ*(*t*) > 0 for *t* > 0. By continuity, ‖*w*^1^(.,*t*) − *w*^2^(.,*t*)‖_2_ = 0 on 0 ≤ *t* ≤ *T*. As shown in [13], backward uniqueness also holds true for the Navier-Stokes equations. This result was obtained by establishing an appropriate stability inequality, similar to Eq. (1), for these equations.

## 3. Backward Continuity and the Hölder Exponent *µ*(*t*)

**Fig. 1:**
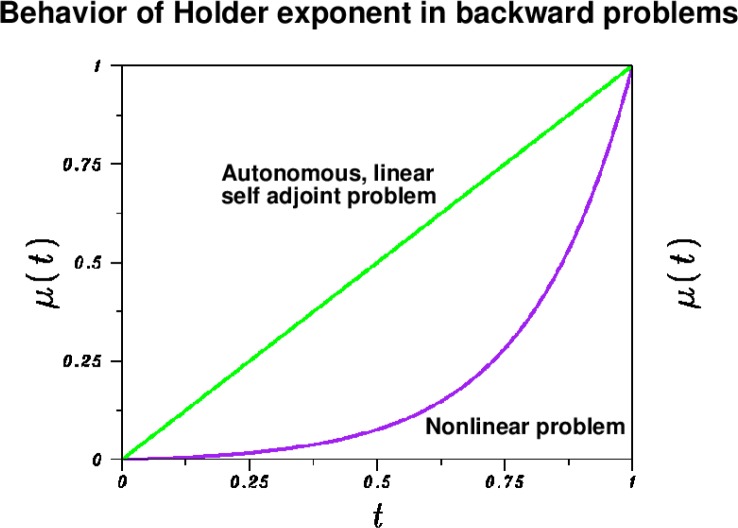
Behavior of Hölder exponent *µ*(*t*) in inequality (4) reflects rate at which the forward evolution equation *w_t_=Lw* has forgotten the past, as *t* increases from *t* = 0 to *t=T* = 1. Deviations away from a linear, time-independent, self adjoint spatial differential operator *L*, can lead to exponential decay in *µ*(*t*), *t* ↓ 0 and affect backward reconstruction from *t=T*.

In many engineering or applied science contexts, only educated guesses would generally be available to estimate *δ* and *M*, rather than exact values. Typically, the *L*^2^ relative error

(5) $${\left\|w(.,T)-f\right\|}_{2}/{\left\|w(.,T)\right\|}_{2}\leq \delta /\left\{{\left\|f\right\|}_{2}-\delta \right\}\approx \delta /{\left\|f\right\|}_{2},$$

might be expected to be on the order of 1% or thereabouts. Since the given data *f* (*x*) may simultaneously approximate several distinct solutions *w^p^*(*x*, *t*) of Eq. (2) at time *T*, there are, in general, infinitely many possible solutions of Eqs. (2) and (3). If *δ* is small, it is generally assumed that any two such solutions would differ only slightly. The extent to which this expectation is justified depends on the decay behavior in the Hölder exponent *µ*(*t*) as illustrated in Fig. 1. In the best possible case, that of a linear self adjoint elliptic operator *L* with time-independent coefficients, we have *µ*(*t*) = *t*/*T*, so that *µ*(*t*) decays *linearly* to zero as continuation progresses from *t=T* to *t* = 0. At *t=T*/2, we have *µ* (*T*/2) = 1/2, and ${\left\|w^{1}\left(.,T/2\right)-w^{2}\left(.,T/2\right)\right\|}_{2}\leq 2\sqrt{M\delta }$. This indicates a loss of acccuracy from *O*(*δ*) to $O(\sqrt{\delta })$, while still only half way to *t* = 0. More typically, *µ*(*t*) is *sublinear* in *t*, possibly with rapid exponential decay. This can lead to much more severe loss of accuracy as reconstruction progresses to *t* = 0. Such rapid decay of *µ* to zero can be brought about by various factors, including nonlinearity, non self adjointness, diffusion coefficients that grow rapidly with time, or adverse spectral properties in the elliptic operator *L*. In all cases, Eq. (4) does not guarantee any accuracy at *t* = 0, but only provides the redundant information ‖*w*^1^(.,0) − *w*^2^(.,0)‖_2_ ≤ 2*M*.

### 3.1 Exponentially Decaying Hölder Exponent

The following is a simple example of a parabolic equation with exponentially decaying *µ*(*t*). With constant *c* > 0, consider the heat conduction problem

(6) $$w_{t}=\exp(ct)w_{\mathrm{xx}},0<x<\pi ,t>0,w_{x}(0,t)=w_{x}(\pi ,t)=0,t\geq 0,$$

with initial values *w*(*x*,0) = cos *x*, 0 ≤ *x* ≤*π*. This has the unique solution

(7) $$w(x,t)=\exp\{(1-e^{\mathrm{ct}})/c\}\cos x,t\geq 0.$$

let With fixed *T* > 0, let *µ*(*t*) = {1− exp(*ct*)}/{1−exp(*cT*)}, 0 ≤ *t* ≤ *T*. Then

(8) $${\left\|w(.,t)\right\|}_{2}={\left\|w(.,0)\right\|}_{2}^{1-\mu (t)}{\left\|w(.,T)\right\|}_{2}^{\mu (t)},\;0\leq t\leq T.$$

In this linear self adjoint problem with growing time-dependent diffusion coefficient, *µ*(*t*) decays exponentially to zero as *t* ↓ 0, with faster decay the larger the value of *c* > 0 in Eq. (6). Here, even *low frequency* information may be unrecoverable backward in time, despite highly accurate data at time *T* > 0. Thus, if *c* = 5, the *smooth and non-negative* solution *w*^†^(*x*, *t*) = 1.0 + exp{(1−*e^ct^*)/*c*}cos *x*, 0 ≤ *t* < 1, cannot be recovered from given *T* = 1 continuation data *f* (*x*) ≡ 1.0, even though *f* (*x*) approximates *w*^†^(*x*,1) to within 1.0×10^−12^, pointwise.

### 3.2 Effective Backward Non-Uniqueness in Non Self Adjoint Problem

Reconstructing the correct backward solution from reasonably accurate data at some *T* > 0, can be a major challenge even with slowly varying diffusion coefficients. The following counterexample was discovered computationally, using the parabolic solver methodology described in [6]. It involves a linear non self adjoint equation with variable coefficients, non-negative initial values, and non-negative solution. With *a* =*α* = 0.05, *σ* = 0.025, consider

(9) $$\begin{array}{l} w_{t}=a{\left\{e^{\left(\sigma x+\alpha t\right)}w_{x}\right\}}_{x}+\left\{\sin(4\pi x)\right\}w_{x},-1<x<1,0<t\leq 1.0,\\ w(x,0)=e^{3x}\sin^{2}(3\pi x),-1\leq x\leq 1,\;w(-1,t)=w(1,t)=0,t\geq 0. \end{array}$$

**Fig. 2:**
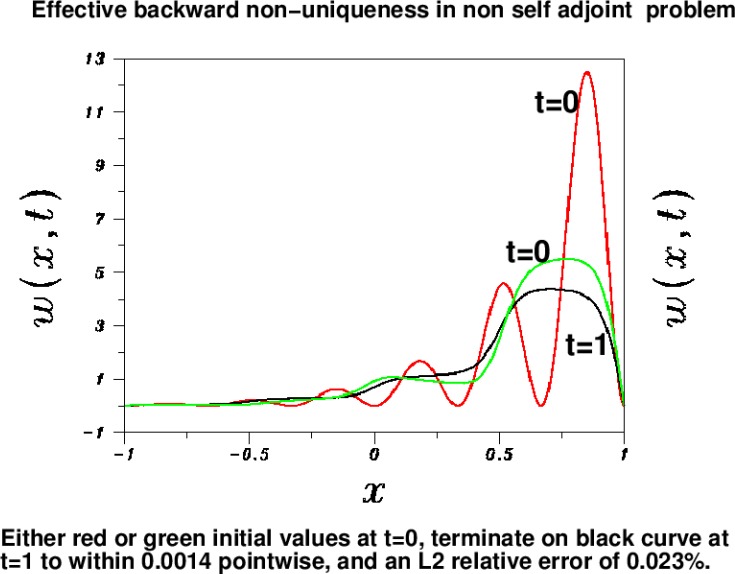
Ill behavior in non self adjoint problem. Accurate data *f* (*x*) (black curve), approximates two distinct solutions *w^red^* (*x*, *t*), (*w^green^ x*, *t*) at time *t* = 1, with an *L*^2^ relative error of 0.023 %, and a pointwise accuracy of 1.4×10^−3^. These solutions originate from the vastly different initial values $w_{0}^{\mathrm{red}}(x)$, and $w_{0}^{green}(x)$.

Let $w_{0}^{\mathrm{red}}(x)$, shown as the red trace in Fig. 2, denote the initial data in Eq. (9), and let *w^red^* (*x*, *t*) be the corresponding solution. An accurate approximation to *w^red^* (*x*, 1) can be obtained numerically by integrating up to time *t* = 1. That approximation, denoted by *f* (*x*) is shown as the black trace in Fig. 2. The green trace in Fig. 2, $w_{0}^{green}(x)$, represents entirely different initial values in Eq. (9). However, the corresponding solution at *t* = 1, *w^green^* (*x*, 1), can also be well-approximated by the black trace *f* (*x*). Indeed, *w^green^* (*x*, 1) agrees with *f* (*x*) to within 1.4×10^−3^ pointwise, with an *L*^2^ relative error of 0.023% Also, ${\left\|w_{0}^{\mathrm{red}}\right\|}_{2}=3.3$, while ${\left\|w_{0}^{green}\right\|}_{2}=2.4$. Therefore, both solutions *w^red^* (*x*, *t*) and *w^green^* (*x*, *t*) satisfy

(10) $${\left\|w(.,1)-f\right\|}_{2}\leq \delta \leq 0.00023{\left\|f\right\|}_{2},\;{\left\|w(.,3)\right\|}_{2}\leq M=3.0.$$

Evidently, quite distinct initial values at *t* = 0 can produce almost identical solutions at *t* =1. This is a good example of effective backward non uniqueness. Indeed, if $w_{0}^{\mathrm{red}}(x)$ is the true solution in this example, the *false* solution $w_{0}^{green}(x)$ would seem to be the more likely initial value, given the black *t* = 1 trace in Fig. 2. In ill-posed inverse problem computations, smoothness and non negativity of solutions are considered beneficial regularizing constraints. Here, both traces are smooth and non negative, satisfy the reasonable *L*^2^ bounds in Eq. (10), and yet the ambiguity remains.

## 4. 2D Nonlinear Parabolic Equations and the Solution Operator Λ*^T^*

The computational experiments discussed below involve four images and two parabolic equations. Numerous other equations can be considered, and a large variety of unexpected phenomena are yet to be uncovered. Let Ω be the unit square 0 < *x*, *y* < 1 in the (*x*, *y*) plane. With fixed *T* > 0, and homogeneous Neumann boundary conditions on ∂Ω, the following initial value problem will be studied,

(11) $$\begin{array}{l} w_{t}=\gamma r(w)\nabla .\{q(x,y,t)\nabla w\}+aww_{x},+b(w_{\cos}{}^{2}w)w_{y},\;\Omega \times (0,T),\\ w(x,y,0)=g(x,y). \end{array}$$

Here *γ* = 8.5×10^−4^, *a, b*, are non negative constants to be prescribed, and

(12) $$\begin{array}{l} r(w)=\exp(0.025w),\\ q(x,y,t)=\exp(10t)(1+5e^{2y}\sin\pi x)\geq 1,\;\Omega \times (0,T). \end{array}$$

An equation with different nonlinearities will also be considered. This is

(13) $$\begin{array}{l} w_{t}=\gamma s(w)\nabla .\{q(x,y,t)\nabla w\}+c\sqrt{\left|w\right|}w_{x},+d(w\cos^{2}w)w_{y},\;\Omega \times (0,T),\\ w(x,y,0)=g(x,y), \end{array}$$

With *γ* and *q*(*x*, *y*, *t*) as in Eq. (11), *c*, *d*, non negative constants to be prescribed, and

(14) $$s(w)=1.0+0.00125w^{2}.$$

Each of Eqs. (11), (13), is well posed in *L*^2^(Ω). Accordingly, given any initial value *w*(*x*, *y*, 0) = *g*(*x*, *y*) ∈ *L*^2^(Ω), a unique solution *w*(*x*, *y*, *T*) exists at time *T*, and the solution operator Λ*^T^*, where

(15) $$\Lambda^{T}w(x,y,0)=w(x,y,T),$$

is well-defined on *L*^2^(Ω). The nonlinear operator is Λ*^T^* not known explicitly. Rather, Λ*^T^ w*(*x*, *y*, 0) must be found by solving the appropriate initial value problem Eq. (11), or Eq. (13), and obtaining the corresponding solution at time *T*. Note that *w*(*x*, *y*, *T*) necessarily belongs to a very restricted class of smooth functions.

In the image deblurring experiments in Sec. 6, Eq. (11) will be used to blur the sharp MRI brain image (image A), and the sharp Marylin Monroe image (image D), by using these images as the initial data *g*(*x*, *y*). The sharp USS Eisenhower image (image G), and the sharp Sydney Opera House image (imageJ), will be blurred using Eq. (13).

## 5. Continuation Backward in Time and the Van Cittert Iteration

In its original formulation, given the data *f* (*x*) and the explicitly known 1D linear convolution integral operator for the unknown *S* with Fourier transform Ŝ(*ω*) > 0, the Van Cittert method solves *Sg=f* for the unknown *Sg*(*x*) by means of the iterative procedure

(16) $$h^{m+1}(x)=h^{m}(x)+\lambda \{f(x)-S[h^{m}(x)]\},\;m\geq 1.$$

Here, *λ* > 0, is a fixed relaxation parameter chosen so that 1 − *λ Ŝ*(*ω*) > 0, *h*^1^ (*x*) = *λ f* (*x*) and the expectation is that *h^m^* → *g*. In fact, in spectroscopy and image processing applications [11,14], the Van Cittert method generally produces useful results after finitely many iterations, even though it may not converge.

We consider using this in the present parabolic context to recover *w*(*x*, *y*, 0) = *g*(*x*, *y*) in Eqs. (11) and (13), given approximate values *f* (*x*, *y*) for the true solution *w*(*x*, *y*, *T*) at time *T*. This requires solving Λ*^T^ g*(*x*, *y*) = *f* (*x*, *y*), using the iterative procedure

(17) $$h^{m+1}(x,y)=h^{m}(x,y)+\lambda \{f(x,y)-\Lambda^{T}h^{m}(x,y)\},\;m\geq 1,$$

with some fixed λ such that 0 < λ < 1, and (*x*, *y*) *h*^1^= λ*f* (*x*, *y*). Clearly, in the present parabolic context, the Van Cittert iteration is unlikely to converge. Indeed, if *h^m^* → *h*^†^ in *L*^2^(Ω) in Eq. (17), then Λ*^T^ h*^†^(*x*, *y*) = *f* (*x*, *y*). However, Λ*^T^ h*^†^(*x*, *y*) satisfies restrictive smoothness requirements, and these are not likely to be met by the approximate data *f* (*x*, *y*). In addition, the nonlinear operator Λ*^T^* in Eq. (17) bears little resemblance to the linear convolution operator *S* in Eq. (16). Nevertheless, remarkably, the Van Cittert iteration is found to be a valuable tool in a wide variety of 2D nonlinear backward parabolic equations. In many cases, this procedure generates iterates *h^m^* (*x*, *y*) such that the *L*^∞^ norm of the residual, ‖ *f* − Λ*^T^ h^m^* ‖_∞_, decays quasi-monotonically to a reasonably small value after a finite number *N* of iterations, and *h^N^* (*x*, *y*) is found to be a useful approximation to *w*(*x*, *y*, 0).

As noted in Sec. 3, backward continuation in certain classes of parabolic equations can be especially challenging. Accordingly, interesting continuation problems may exist where the procedure in Eq. (17) cannot produce useful results.

### 5.1 Explicit Finite Difference Scheme for Computing Λ*^T^ w*(*x*, *y*, 0)

A convenient and effective numerical procedure for solving the nonlinear initial value problems in Eqs. (11) and (13), is based on finite differences, using explicit time diffencing and centered space differencing. This leads to modest *O*(Δ*t* + (Δ*x*)^2^ + (Δ*y*)^2^) accuracy. However, the necessary stability condition on Δ*t* for explicit schemes, improves that accuracy to *O*((Δ*x*)^2^ + (Δ*y*)^2^). Higher precision numerics, together with higher resolution imagery, will be considered in subsequent reports. This paper deals with 8 bit gray scale 256× 256 pixel images, with pixel values ranging between 0 and 255. With Δ*x* = Δ*y* = 1/256, Δ*t* = 3.0×10^−7^, the following difference approximation is used to march the discrete mesh function *W^n^* ≡ *W*(*j*Δ*x*, *k*Δ*y*, *n*Δ*t*) in Eq. (11), 400 time steps Δ*t* forward in time, up to time *T* = 1.2×10^−4^,

(18) $$\begin{array}{l} W^{n+1}=W^{n}+\Delta t\gamma R(W^{n})\nabla .\{Q^{n}\nabla W^{n}\}+aW^{n}W_{x}^{n}\\ \;+b(W^{n}\cos^{2}W^{n})W_{y}^{n},\;n=0,399,\\ \;W^{0}=g(x,y). \end{array}$$

Homogeneous Neumann conditions are applied on the boundary of the unit square. The same mesh parameters and finite differencing are used for blurring with Eq. (13). In this notation, *W*^0^ denotes the original sharp image *g*(*x*, *y*), while *W*^400^ is the nonlinearly blurred image *f* (*x*, *y*) using either Eq. (11) or, Eq. (13). Define the discrete nonlinear parabolic blurring operator $\Lambda_{d}^{T}$ by

(19) $$\Lambda_{d}^{T}W^{0}=W^{400}.$$

This nonlinear operator is defined on any 8 bit gray scale 256×256 pixel image *g*(*x*, *y*). Applying $\Lambda_{d}^{T}$ to that image simply means applying the above explicit scheme for 400 time steps to *W*^0^, and acquiring the resulting array *f* (*x*, *y*) = *W*^400^. We stress that the blurred image *f* (*x*, *y*) so obtained is only an approximation to the true solution *w*(*x*, *y*, *T*) in Eq. (11) or Eq. (13).

Image diagnostic statistical information will use the discrete *L*^1^, *L*^2^, and total variation norms, defined by

(20) $${\left\|f\right\|}_{p}={\left\{{\left(256\right)}^{-2}\sum_{j,k=1}^{256}{\left|f(x_{j},y_{k})\right|}^{p}\right\}}^{1/p},\;p=1,2,$$

and

(21) $${\left\|f\right\|}_{\mathrm{TV}}|\equiv {\left\|\nabla f\right\|}_{1}={\left(256\right)}^{-2}\sum_{j,k=1}^{255}{\left({\left\{f^{x}(x_{j},y_{k})\right\}}^{2}+{\left\{f^{y}(x_{j},y_{k})\right\}}^{2}\right)}^{1/2},$$

where

(22) $$\begin{array}{l} f^{x}(x_{j},y_{j})={\left(256\right)}^{-1}\left(f(x_{j+1},y_{k})-f(x_{j},y_{k})\right),\\ f^{y}(x_{j},y_{k})={\left(256\right)}^{-1}\left(f(x_{j},y_{k+1})-f(x_{j},y_{k})\right). \end{array}$$

In addition, the *peak signal to noise ratio* (*PSNR*), will be used as an image quality metric. If *g*(*x*, *y*) is the original sharp image, and *f* (*x*, *y*) is any degraded version of *g*(*x*, *y*), this is defined by

(23) $$PSNR=-20\log_{10}\left\{{\left\|f-g\right\|}_{2}/255\right\}.$$

## 6. Nonlinear Blurring and Deblurring Experiments

Very little seems to be currently known regarding backward in time continuation in multidimensional nonlinear parabolic equations, and the experiments described below, involving relatively simple nonlinearities, already represent uncharted waters. An important advantage of the Van Cittert method is the ‘self regularizing’ property of the iterative process, whereby low frequency information is reconstructed in the first few iterations, while many more iterations are needed to acquire high frequency information. Several other iterative restoration methods have this property. As a consequence, useful information can often be retrieved by stopping the iterative process before amplification of high frequency noise overwhelms the reconstruction.

The results developed in Sec. 3, concerning backward stability and the Hölder exponent *µ*(*t*), will inform the subsequent discussion. While backward uniqueness is characteristic of a large class of linear and nonlinear parabolic equations, the major practical difficulty lies in recovering the correct solution from the limited precision available in the given continuation data. Deblurring nonlinearly blurred imagery involves the recovery of fairly complex initial data at time *t* = 0, by nonlinear backward continuation of imprecise data at some *T* > 0. In fact, as is typically the case in applications, the accuracy in the blurred image data *f* (*x*, *y*) = *W*^400^ in Eq. (18), as an approximation to the elusive true solution *w*(*x*, *y*, *T*) in Eq. (11) or Eq. (13), is actually *unknown*.

In addition, Eqs. (11) and (13) are strongly nonlinear through the functions *r*(*w*) and *s*(*w*), with *w*(*x*, *y*, *t*) ranging between 0 and 255. Moreover, there is the space and time dependent function *q*(*x*, *y*, *t*), and the terms in *ww_x_* and *ww_y_*. Such equations deviate strongly from the autonomous, linear, self adjoint case, for which substantial computational experience exists. While a stability inequality such as Eq. (4) can be derived for each of Eqs. (11) and (13), the resulting functional form for the Hölder exponent *µ*(*t*) is unlikely to be precise. In summary, neither *δ* nor *µ*(*t*) are likely to be known in the stability estimate ‖*w*^1^(.,*t*) − *w*^2^(.,*t*) ‖_2_ ≤ 2*M*^1−^*^µ^*^(^*^t^*^)^*δ^µ^*^(^*^t^*^)^, 0 ≤ *t* ≤ *T*, for either of Eqs. (11) or (13).

The results in Figs. 1 and 2, together with the examples in [6], indicate that only a modest degree of success can be expected in backward continuation in Eqs. (11) and (13). In the present paper, knowledge of the original sharp image can be used to gauge the usefulness of the deblurred image produced by backward continuation. However, in applications unrelated to imaging, using field data of unknown precision, the degree of success or failure in nonlinear backward continuation may not be as easily ascertained. As shown in [6], there is the possibility of producing a smooth, physically plausible, yet *false* reconstruction.

### 6.1 MRI Brain Image

**Fig. 3:**
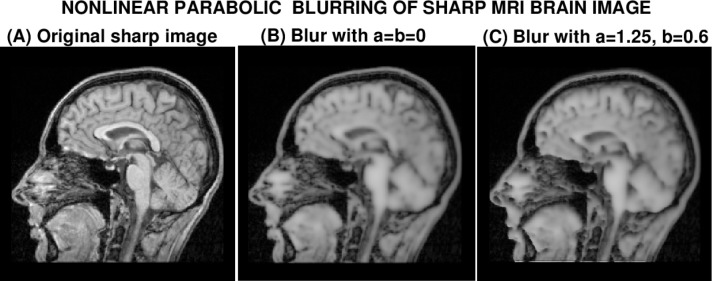
Nonlinear parabolic blurring of sharp MRI brain image *g*(*x*, *y*), by using it as initial values in Eq. (11) with two different sets of values for the constants *a*,*b*.

In Fig. 3, the original sharp MRI brain image (A) is blurred to form image (B), by applying the finite difference scheme in Eq. (18) to the parabolic equation Eq. (11), with coefficients *a=b* = 0. A different blurred image is then obtained, image (C), by repeating this process with coefficients *a* = 1.25, *b* = 0.6. Images (B) and (C) appear very similar in quality, and, from Table 1, both these images have almost the same values for ‖*f*‖_1_, ‖*f*‖_2_ and ‖∇*f*‖_1_. In particular, ‖∇*f*‖_1_ has been reduced by almost a factor of two from its original value in image (A), reflecting substantial blurring. The *PSNR* value in image (C) is noticeably smaller than in image (B), indicating greater degradation in image (C). However, since the *PSNR* metric requires knowledge of the original sharp image, in practice, such increased degradation in image (C) would not be known to a user. In fact, both images (B) and (C) appear to have been blurred, more or less equally, by convolution with a type of Gaussian-like point spread function.

**Fig. 4:**
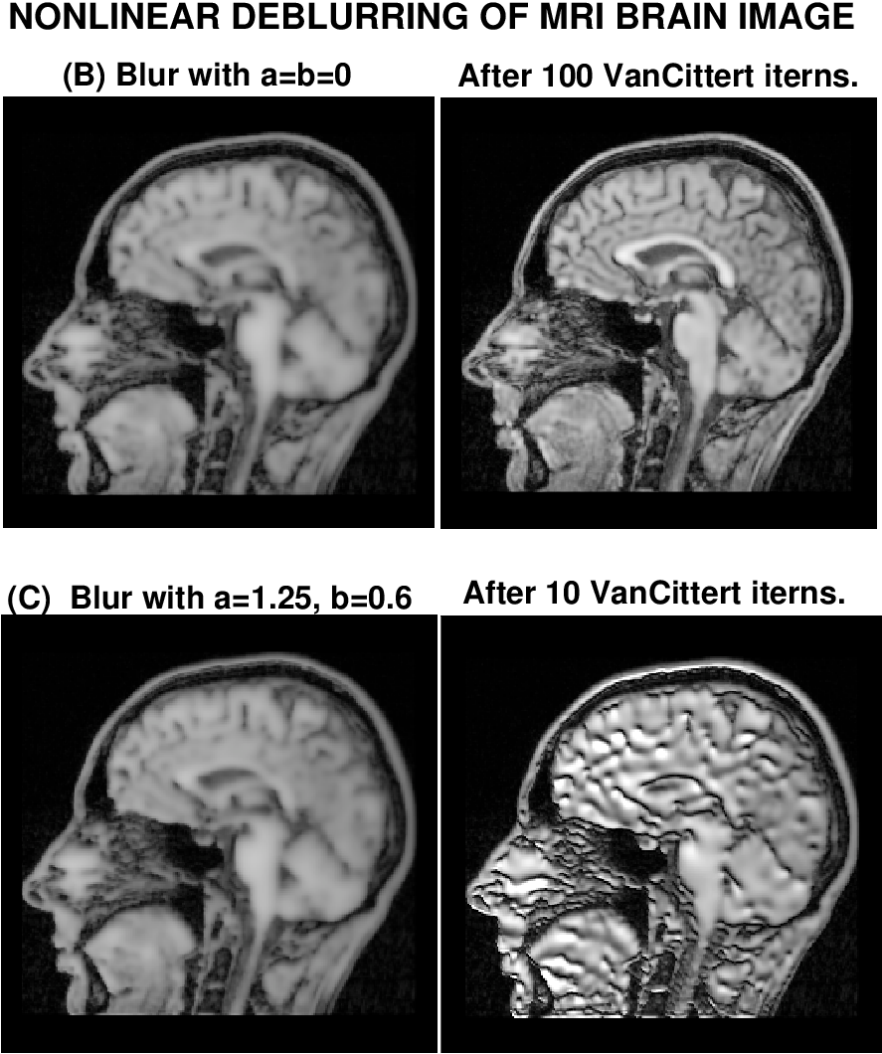
Nonlinearly blurred image (B) was successfully deblurred after 100 iterations. Visually similar image (C), blurred with additional nonlinearities, could not be deblurred.

Figure 4 displays the results of backward in time continuation in Eq. (11), using the Van Cittert iteration in Eq. (17), with $\Lambda_{d}^{T}$ as in Eq. (19), and *λ* = 0.5. Remarkably, despite the strongly nonlinear blurring in image (B) through the function *r*(*w*) in Eq. (12), useful deblurring of that image is obtained after 100 iterations. From Table 2, we see that the values of‖*f*‖_1_ and ‖*f*‖_2_ in the deblurred image (B), are very close to their original values in image (A), while ‖∇*f*‖_1_ has recovered almost 90 % of its original value. Also, deblurring in image (B) has increased the *PSNR* from 25 to 34.

The results of deblurring image (C), shown in in Fig. 4, are sharply different, and unexpected. After 10 Van Cittert iterations, using *a* = 1.25, *b* = 0.6, in Eq. (18), most useful information has been lost in the deblurred image, and this without explosive noise amplification. Indeed, in Table 2, the values for ‖*f*‖_1_ and ‖*f*‖_2_ after 10 iterations are about 12 % larger than their true values in the original image (A), well within the range of what might have been prescribed to stabilize continuation. One possible explanation is that the inclusion of the terms in *ww_x_* and *ww_y_* in Eq. (11) renders backward stability more precarious, (see Fig. 1), and the accuracy in the continuation data represented by image (C) is no longer sufficient to recover the sharp image.

### 6.2 Marilyn Monroe Image

**Fig. 5:**
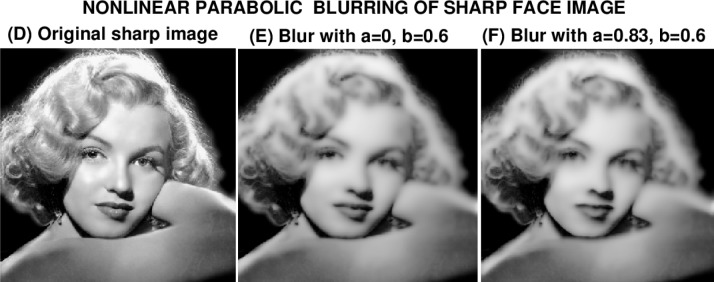
Nonlinear parabolic blurring of sharp Marilyn Monroe image *g*(*x*, *y*), by using it as initial values in Eq. (11) with two different sets of values for the constants *a*, *b*.

**Fig. 6:**
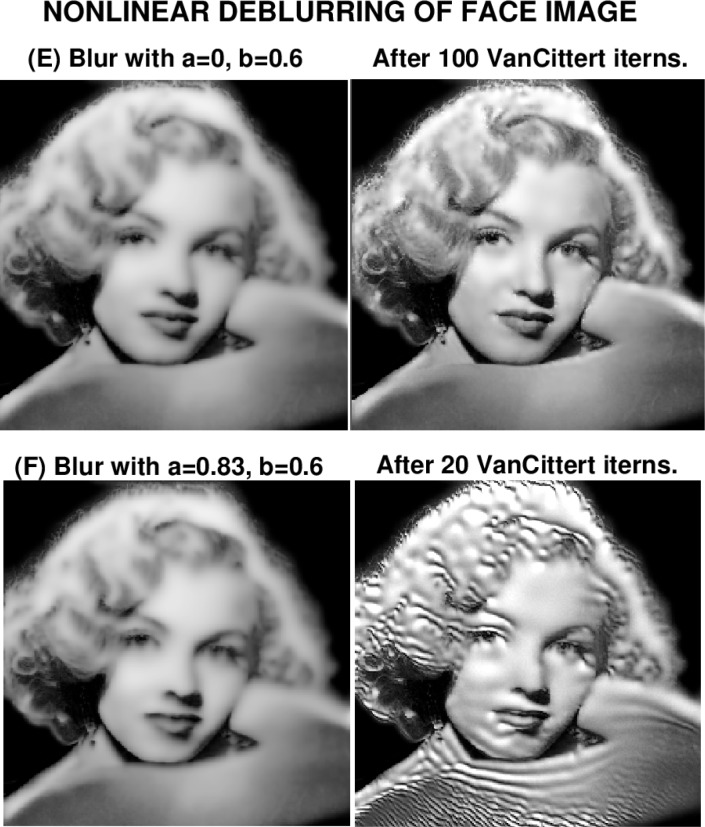
Nonlinearly blurred image (E) was succesfully deblurred after 100 iterations. Visually similar image (F), blurred with additional nonlinearities, could not be usefully deblurred.

In view of the unexpected failure in deblurring image (C), the experiments in Figs. 5 and 6 aim at elucidating the influence of the nonlinear terms involving *ww_x_* and *ww_y_*, on backward continuation in Eq. (11). Since the *ww_y_* term is modulated by the factor cos^2^*w* in Eq. (11), it may not be as destabilizing as the *ww_x_* term. Accordingly, the sharp Marilyn Monroe image (D) is first blurred using Eq. (18) with *a* = 0, *b* = 0.6, to form the blurred image (E). The process is then repeated with *a* = 0.83, *b* = 0.6, to form image (F). In Fig. 5, image (F) differs noticeably from image (E) qualitatively, yet, as shown in Table 3, both these images have almost the same values for ‖*f*‖_1_, ‖*f*‖_2_ and ‖∇*f*‖_1_. However, image (F) has a smaller *PSNR* value. Evidently, the *ww_x_* term in Eq. (11) is responsible for the increased degradation in +image (F).

Figure 6 displays the results of deblurring these two images. Again, remarkably, despite the strongly nonlinear blurring in image (E) through the function *r*(*w*), and the inclusion of the *ww_y_* term in Eq. (11), very good results are obtained for that image, after 100 Van Cittert iterations. As shown in Table 4, the values of ‖*f*‖_1_, and ‖*f*‖_2_ in the deblurred image (E), are very close to their original values, while ‖∇*f*‖_1_ has recovered 83 % of its value in image (D). Also, deblurring in image (E) has increased the *PSNR* from 24 to 29. These improvements are more modest than were achieved in deblurring the MRI brain image (B). However, both the *ww_x_* and *ww_y_* terms in Eq. (11) were excluded in forming the blurred image (B). As was the case in image (C), deblurring image (F) was unsuccessful. After 20 Van Cittert iterations, using *a* = 0.83, *b* = 0.6, in Eq. (18), some sharpening has clearly occurred, but the image is marred by artifacts. Again, there is no high frequency noise amplification in the deblurred image (F), and the values for ‖*f*‖_1_ and ‖*f*‖_2_ after 20 iterations, are about 7 % larger than their true values. Thus, the deblurred image (F) satisfies such *a-priori* bounds as might have been placed to stabilize ill-posed continuation. Clearly, the term in *ww_x_* in Eq. (11) emerges as the prime suspect in misbehaved backward continuation.

### 6.3 USS Eisenhower Image

**Fig. 7:**
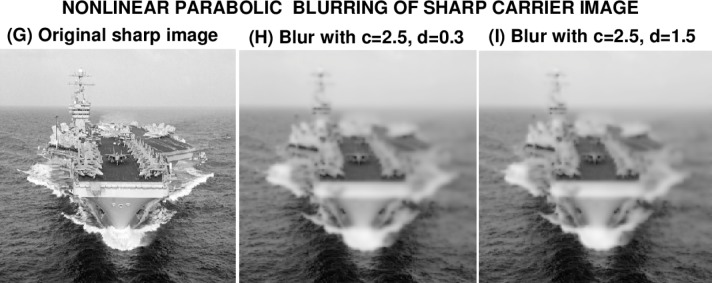
Nonlinear parabolic blurring of sharp USS Eisenhower image *g*(*x*, *y*), by using it as initial values in Eq. (13) with two different sets of values for the constants *c*, *d*.

Computational experiments on the next two images study the results of blurring using Eq. (13), where the milder term $\sqrt{\left|w\right|}w_{x}$ replaces the troublesome term *ww_x_* in Eq. (11). The sharp USS Eisenhower image (G) is first blurred using Eq. (13) with *c* = 2.5, *d* = 0.3, to form the blurred image (H). The process is then repeated with *c* = 2.5, *d* = 1.5, to form image (I). In Fig. 7, image (H) is visually indistinguishable from image (I). Interestingly, as shown in Table 5, and unlike the previous examples in Figs. 3 and 5, images (H) and (I) have the *same PSNR* value, as well as almost the same values for ‖*f*‖_1_, ‖*f*‖_2_ and ‖∇*f*‖_1_. In the present case, there is no metric available that can be used to predict success or failure in deblurring images (H) and (I).

**Fig. 8:**
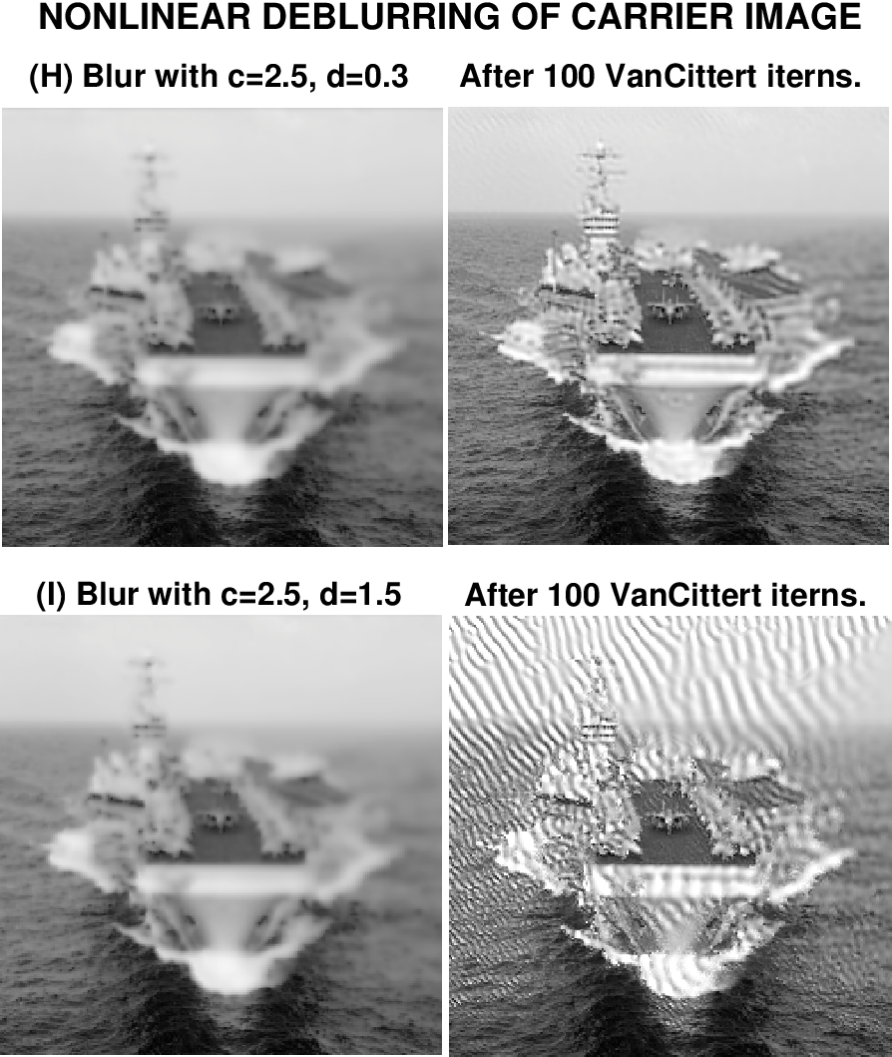
Nonlinearly blurred image (H) was successfully deblurred after 100 iterations. Visually indistinguishable image (I), blurred with stronger nonlinearities, could not be usefully deblurred.

Figure 8 displays the results of deblurring these two images. Despite the strongly nonlinear blurring in image (H), through the function *s*(*w*) and the inclusion of both the $\sqrt{\left|w\right|}w_{x}$ and *ww_y_* terms in Eq. (13), reasonably good results are obtained after 100 Van Cittert iterations. The carrier’s command ‘island’ has been recovered, along with the two rows of planes on deck. As shown in Table 6, the values of ‖*f*‖_1_ and ‖*f*‖_2_ in the deblurred image (H), are very close to their original values, while ‖∇*f*‖_1_ has recovered 78 % of its value in image (G). Also, deblurring in image (H) has increased the *PSNR* from 20 to 23. It is noteworthy that the $2.5\sqrt{\left|w\right|}w_{x}$ term in Eq. (13) did not preclude useful reconstruction in image (H). Surprisingly, deblurring in image (I) was not successful. There is no high frequency noise amplification in the deblurred image (I), even after 100 iterations, and the values of ‖*f*‖_1_ and ‖*f*‖_2_ are only about 3 % higher than their true values in image (G), as shown in Table 6. As was the case in the deblurred Marilyn Monroe image (F), substantial sharpening has occurred in the deblurred image (I), but the sharpened image is seriously marred by artifacts. Because of the moderating effect of the factor cos^2^*w*, it was not anticipated that the term 1.5*w*(cos^2^*w*)*w_y_* in Eq. (13) might be detrimental in image (I), since the term $2.5\sqrt{\left|w\right|}w_{x}$

was well-tolerated in image (H), and, previously, the term 0.6*w*(cos^2^*w*)*w_y_* did not prevent successful deblurring of the Marilyn Monroe image (E). The capricious behavior in image (I) would appear to justify the term *hazardous continuation* used in the title of this paper.

### 6.4 Sydney Opera House Image

**Fig. 9:**
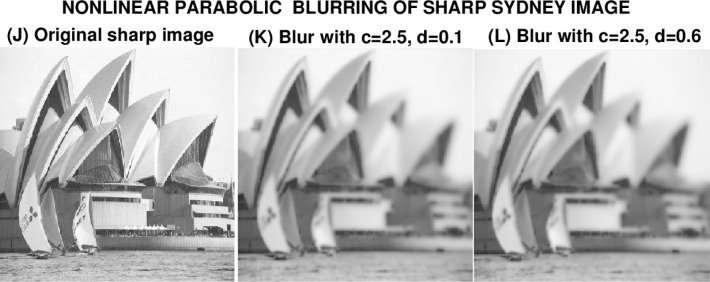
Nonlinear parabolic blurring of sharp Sydney Opera House image *g*(*x*, *y*), by using it as initial values in Eq. (13) with two different sets of values for the constants *c*, *d*.

**Fig. 10:**
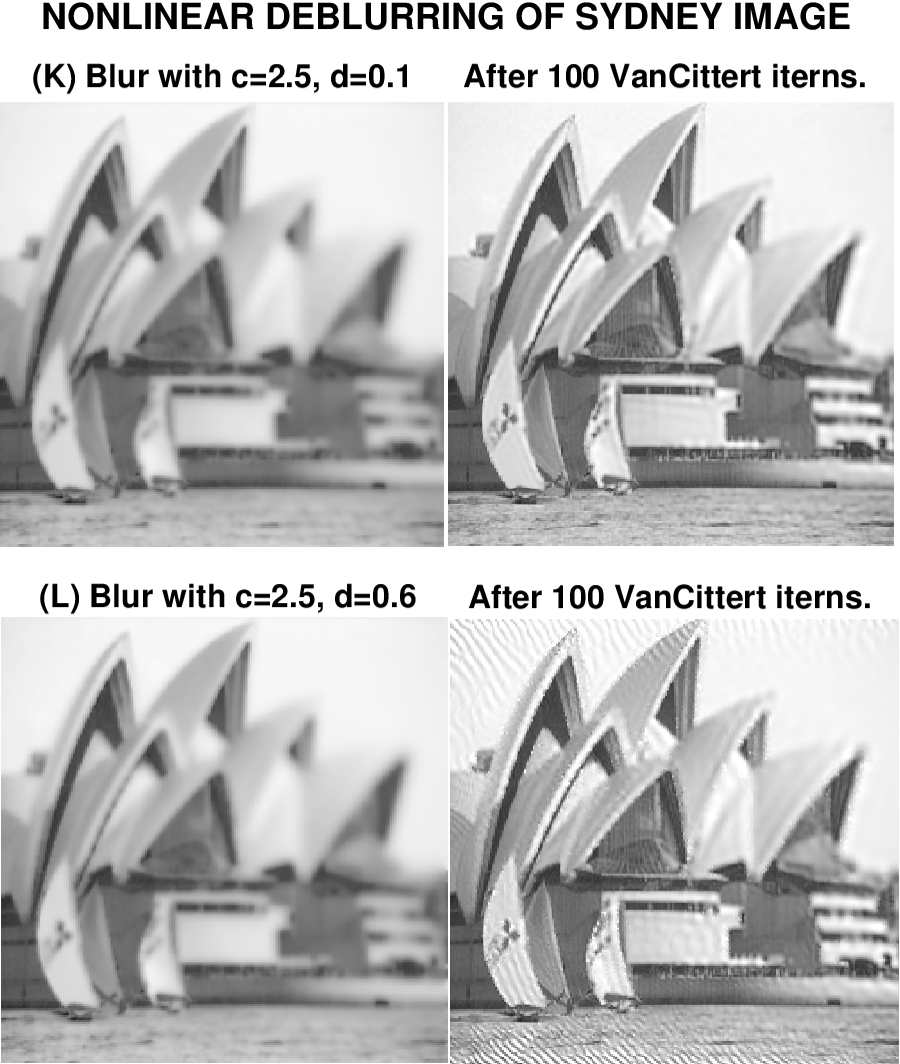
Nonlinearly blurred image (K) was successfully deblurred after 100 iterations. Visually indistinguishable image (L), blurred with stronger nonlinearities, could not be usefully deblurred.

The results in this experiment confirm the unpredictability found in the previous example using Eq. (13), and justify the title even more strongly. Here, the coefficient *d* multiplying the *w*(_cos_^2^*w*)*w_y_* term was substantially reduced. Again, in Fig. 9, images (K) and (L) are visually and statistically indistinguishable, with the same *PSNR* value, and almost the same values for ‖*f*‖_1_, ‖*f*‖_2_ and ‖∇*f*‖_1_. In Fig. 10, image (K) with *c* = 2.5, = *d* = 0.1 is successfully deblurred, and the *PSNR* value has increased from 19 to 23. In image (L), where *d* = 0.6, there is visible sharpening, with the *PSNR* increasing from 19 to 21. However, the sharpened image is again marred by artifacts. There is no high frequency noise amplification, even after 100 iterations, and the values of ‖*f*‖_1_ and ‖*f*‖_2_ in the deblurred image (L), are little changed from their true values in image (J). Again, inexplicably, while the term $2.5\sqrt{\left|w\right|}w_{x}$ in Eq. (13) was tolerated in image (L), and the term 0.6*w*(_cos_^2^*w*)*w_y_* was acceptable in image (E), this same term 0.6*w*(cos^2^*w*)*w_y_* was found troublesome in image (L).

## 7. Concluding Remarks

The successful deblurring of images (B), (E), (H), and (K), indicate the Van Cittert iterative procedure to be a useful tool for backward in time continuation in an important class of 2D nonlinear parabolic equations. A wide variety of nonlinear problems remains to be explored. Surprisingly, the simple nonlinearities in Eqs. (11) and (13) involving the terms in *ww_x_* and *ww_y_*, were found to be potentially destabilizing, and capable of preventing useful continuation. The practical difficulty of reconstructing the correct backward solution, using data of limited and *unknown* precision, was stressed. Other limitations include the fact that the fundamental stability inequality governing a particular continuation, Eq. (4), can seldom be obtained with sufficient precision. In particular, the rate at which the Hölder exponent *µ*(*t*) tends to zero as *t* ↓ 0, which is of vital interest, is typically unknown. The *unsuccessful* deblurring in images (C), (F), (I), and (L), is of major interest. Visually, the amount of blurring in each of these images is no greater than in the successfully deblurred companion image, and the corresponding values of ‖∇*f*‖_1_ are almost equal in every case. Evidently, how the image was blurred is critical, not just the amount of blur. These failed continuations suggest that the presence of *ww_x_* and *ww_y_* terms in Eqs. (11) and (13), with relatively large coefficients, unexpectedly leads to faster decaying Hölder exponents *µ*(*t*), such as is shown in Fig. 1, and the accuracy in the blurred image data becomes insufficient for useful continuation.

## Figures and Tables

**Table 1:** Behavior using Eq. (11) in nonlinear blurring in Fig. 3.

Image *f* (*x*, *y*)	Parameters *a*, *b*	‖*f*‖_1_	‖*f*‖_2_	‖∇*f*‖_1_	*PSNR*
Sharp image A	Not blurred	59	86	3360	∞
Blurred image B	*a* = 0, *b* = 0	55	78	1740	25
Blurred image C	*a* = 1.25, *b* = 0.6	55	78	1770	20

**Table 2:** Behavior using Eq. (11) in nonlinear deblurring in Fig. 4.

Image *f* (*x*, *y*)	Parameters *a*, *b*	‖*f*‖_1_	‖*f*‖_2_	‖∇*f*‖_1_	*PSNR*
Deblurred image B	*a* = 0, *b* = 0	59	85	2980	34
Deblurred image C	*a* = 1.25, *b* = 0.6	63	96	4680	17

**Table 3:** Behavior using Eq. (11) in nonlinear blurring in Fig. 5.

Image *f* (*x*, *y*)	Parameters *a*, *b*	‖*f*‖_1_	‖*f*‖_2_	‖∇*f*‖_1_	*PSNR*
Sharp image D	Not blurred	107	130	3100	∞
Blurred image E	*a* = 0, *b* = 0.6	101	122	1580	24
Blurred image F	*a* = 0.83, *b* = 0.6	101	122	1550	20

**Table 4:** Behavior using Eq. (11) in nonlinear deblurring in Fig. 6.

Image *f* (*x*, *y*)	Parameters *a*, *b*	‖*f*‖_1_	‖*f*‖_2_	‖∇*f*‖_1_	*PSNR*
Deblurred image E	*a* = 0, *b* = 0.6	106	129	2580	29
Deblurred image F	*a* = 0.83, *b* = 0.6	112	139	5800	18

**Table 5:** Behavior using Eq. (13) in nonlinear blurring in Fig. 7.

Image *f* (*x*, *y*)	Parameters *c*, *d*	‖*f*‖_1_	‖*f*‖_2_	‖∇*f*‖_1_	*PSNR*
Sharp image G	Not blurred	139	153	4760	∞
Blurred image H	*c* = 2.5, *d* = 0.3	134	147	1720	20
Blurred image I	*c* = 2.5, *d* = 1.5	134	148	1770	20

**Table 6:** Behavior using Eq. (13) in nonlinear deblurring in Fig. 8.

Image *f* (*x*, *y*)	Parameters *c*, *d*	‖*f*‖_1_	‖*f*‖_2_	‖∇*f*‖_1_	*PSNR*
Deblurred image H	*c* = 2.5, *d* = 0.3	137	152	3700	23
Deblurred image I	*c* = 2.5, *d* = 1.5	141	157	7700	18

**Table 7:** Behavior using Eq. (13) in nonlinear blurring in Fig. 9.

Image *f* (*x*, *y*)	Parameters *c*, *d*	‖*f*‖_1_	‖*f*‖_2_	‖∇*f*‖_1_	*PSNR*
Sharp image J	Not blurred	173	183	4090	∞
Blurred image K	*c* = 2.5, *d* = 0.1	166	176	1840	19
Blurred image L	*c* = 2.5, *d* =0.6	167	176	1880	19

**Table 8:** Behavior using Eq. (13) in nonlinear deblurring in Fig. 10.

Image *f* (*x*, *y*)	Parameters *c*, *d*	‖*f*‖_1_	‖*f*‖_2_	‖∇*f*‖_1_	*PSNR*
Deblurred image K	*c* = 2.5, *d* = 0.1	171	182	3500	23
Deblurred image L	*c* = 2.5, *d* = 0.6	172	183	4920	21
